# The Relationship between Hematological Indices and Autoimmune Rheumatic Diseases (ARDs), a Meta-Analysis

**DOI:** 10.1038/s41598-017-11398-4

**Published:** 2017-09-07

**Authors:** Xuanyu Hao, Dongyang Li, Dan Wu, Ning Zhang

**Affiliations:** 1 The Second Clinical Academy of China Medical University, Shenyang, Liaoning China; 2 The Department of Rheumatology at Shengjing Hospital of China Medical University, Shenyang, Liaoning China

## Abstract

This meta-analysis was undertaken to investigate the relationship between hematological indices and autoimmune rheumatic diseases (ARDs). PubMed, Embase, and Web of Science were searchedfor studies of ARDs and hematological indices. Standardized mean difference (SMD) was calculated with confidence interval (CI) of 95%. 18 studies were included in our meta-analysis. Compared to the healthy control group, neutrophil–lymphocyte ratio (NLR) was increased in patients with ankylosing spongdylitis(AS), Behçet’s disease(BD), andrheumatoid arthritis(RA)(SMD = 0.33; 95% CI: 0.19 to 0.47; SMD = 1.90; 95% CI: 0.13 to 3.67; SMD = 0.75; 95% CI: 0.23 to 1.28). Platelet–lymphocyte ratio (PLR) was found increased in RA and SLE (SMD = 33.91; 95% CI: 20.50 to 47.32; SMD = 59.11; 95% CI: 4.46 to 113.76). Mean platelet volume (MPV)was irrelevant to BD and SLE by comparing with the healthy control group respectively. (SMD = 0.24; 95% CI: −0.49 to 0.97; SMD = −0.15; 95% CI: −1.77 to 1.48). Red cell distribution width (RDW) was not related to AS (SMD = 0.59, 95% CI: −0.37, 1.55). Our findings indicated that NLR had a strong association with AS, BD, and RA. PLR was also related to RA and SLE. NLR and PLR could be recommended as inexpensive diagnostic biomarkers for ARDs.

## Introduction

Autoimmune rheumatic diseases (ARDs) are chronic autoimmune diseases affecting joints, bones, muscles, skin, cartilage, tendons, ligaments and share features like arthralgia and arthritis, myalgia, and internal organs, such as kidney, lung, and nerves resulting from dysfunction of the immune system regulation and persistent inflammation^1, 2^. ARDs include abroad spectrum of diseases which have impact on patients’ movement and function, involving rheumatoid arthritis(RA), systemic lupus erythematosus(SLE), Ankylosing Spongdylitis(AS), Sjögren’s syndrome(SS), Behçet’s disease(BD), and systemic sclerosis(SSc)^3^.

Chronic inflammation which is triggered by overproduction of autoantibodies, inflammatory cytokine release, and immune complex deposition isa critical pathological manifestation in the disease development process of ARDs, and inflammationlead to the changes on one or more cellular lineages of the hematopoietic system^4, 5^. Thus, hematologic abnormalities play a significant role in the progress of ARDs. C-reactive protein (CRP) and the erythrocyte sedimentation rate (ESR) are most commonly used as markers for the inflammatory response status in patients with RA, SLE, AS and other ARDs^6–10^. Recently, reports on neutrophil–lymphocyte ratio (NLR), platelet–lymphocyte ratio (PLR), mean platelet volume (MPV), and red blood cell distribution width (RDW) show that new indicators have come into use as markers for systemic inflammation. The NLR is the proportion of absolute neutrophil count to lymphocyte count, which has been evaluated in a number of studies involving malignancies, familial Mediterranean fever, cardiovascular diseases, and some rheumatic diseases, however, the relevance between NLR and ARDs is still controversial^4, 7, 11–14^. Several published studies on PLR have been undertaken to show that PLR may relate to ARDs^4, 11, 15–18^. Many recent researches have also focused on whether MPVis increased in patients with rheumatic diseases^4, 19–21^. RDW, which is commonly regarded as a marker for anemia diagnosis is a numerical measurement of the heterogeneity in size of circulating erythrocytes. Some previous studies suggested an association between RDW and rheumatic diseases like AS and SS but some draw the contradictory conclusion^7, 16, 22^. The association between hematological indices and ARDs remains controversial. Therefore, this meta-analysis was conducted to reveal the relationship between hematological indices and ARDs.

## Results

### Included studies

**Figure 1:**
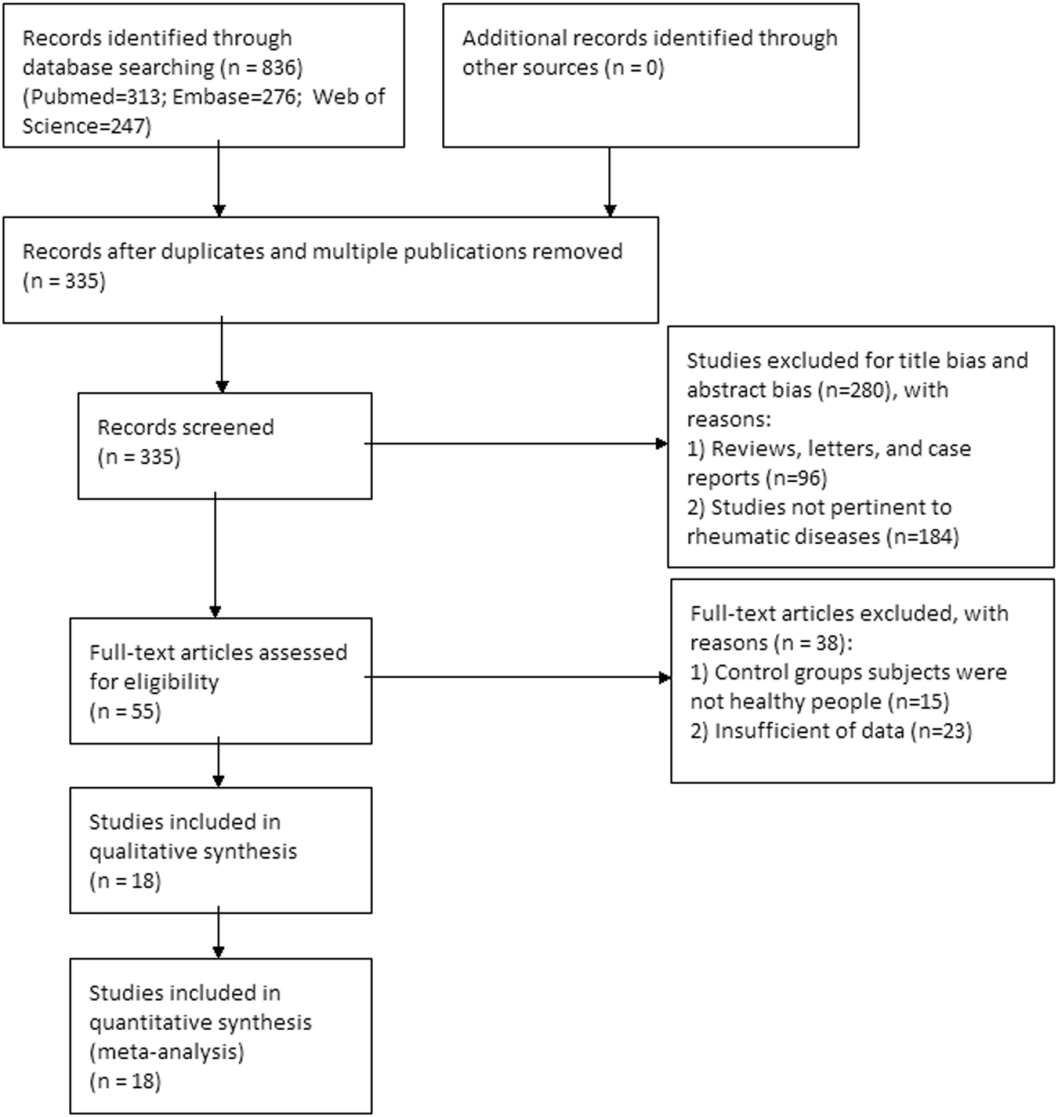
Flow chart of literature search and study selection.

A flowchart diagram of the article selection process was shown in Fig. 1A total of 836 records were obtained after searching for Pubmed, Embase, and Web of Science; of these, 501 duplicates were removed, and 280 articles were excluded after reviewing the title and abstract. Full-texts of remaining 55 studies were retrieved for further review. Among them, 15 articles were excluded since control groups subjects were not healthy people, and 23 articles were excluded because of insufficient of data. Finally, 18 studies were included in the meta-analysis.

### Characteristics of included studies and methodological quality

A total of 18 published articles finally included in this meta-analysis, which were published from 2010 to 2016. The number of included subjects in the patients group and healthy control group ranged from 30 to 317and 25 to 149, respectively. Only 1 study was designed as cross sectional study, other studies were retrospective studies. Of the 18 studies, 17 reported NLR (6 includedpatients with AS, 3 includedpatients with RA, 4 includedpatients with BD, 4 includedpatients with SLE), 7 reported PLR (3 includedpatients with AS, 2 includedpatients with RA, 2 includedpatients with SLE), 5 reported MPV (3 includedpatients with BD, 2 includedpatients with SLE), 3 reported RDW (All 3 studiesincluded patients with AS). The characteristics of included studieswere summarized in Table 1. Supplementary Table S2 presents the assessment of 18 studies. The maximum score was 8 with the NOS scale (n = 15), while the minimum score was 6 (n = 1), and two studies scored 7(see Supplementary Table S2).

**Table 1:** General characteristic of the included studies.

First Author	publication year	study design	types of ARDs	disease duration (mean ± SD)	number		age (mean ± SD)		sex (M/F)		NOS scale
					Patint	Control	Patient	Control	Patient	Control	
A. Balkarl	2016	retrospective study	BD	N	120	79	37.83 ± 11.86	37.63 ± 10.82	72/48	46/33	8
Acikgoz, N.	2010	retrospective study	BD	9.1 ± 5.1	60	40	40.5 ± 12.9	41.3 ± 6.8	31/29	18/22	8
Boyraz, I.	2014	retrospective study	AS	N	105	50	46.83 ± 14.94	41.02 ± 17.37	78/27	28/22	8
Boyraz, I.	2016	retrospective study	AS	8.8 ± 8	30	25	38.6 ± 8.3	37.1 ± 9.4	19/11	16/9	8
Bozan, N.	2016	retrospective study	AS	5.29 ± 5.09	30	35	32 ± 8	32 ± 6	18/12	22/13	7
Coskun, B. N.	2014	retrospective study	AS	9.25 ± 8.37	35	38	38.91 ± 11.90	37.36 ± 6.90	26/9	26/12	8
Gokmen, F.	2015	retrospective study	AS	N	96	81	43.8 ± 12.9	46.5 ± 11.2	65/31	47/34	6
Li, L.	2015	retrospective study	SLE	N	59	149	29.47 ± 12.63	28.44 ± 4.42	4/55	17/132	8
Mercan, R.	2016	retrospective study	AS	N	140	117	37.1 ± 9.8	37.2 ± 13.2	79/61	42/75	8
			RA	N	136	117	51.7 ± 13.5	37.2 ± 13.2	15/121	42/75	
Oehadian, A.	2013	cross sectional study	SLE	N	21	30	28.5 ± 41.08	28.5 ± 60.42	0/21	10/20	8
Ozturk, C.	2015	retrospective study	BD	9.26 ± 7.09	65	62	36.30 ± 10.98	34.16 ± 9.33	38/27	31/31	8
Peng, Y. F.	2014	retrospective study	AS	N	44	113	35.98 ± 16.24	33.87 ± 7.88	30/14	82/31	8
Rifaioglu, E. N.	2014	retrospective study	BD	N	65	136	37.7 ± 12.3	38.8 ± 11.3	38/17	56/80	8
Safak, S.	2014	retrospective study	SLE	N	44	44	42 ± 16	41 ± 17	11/33	9/35	8
Soydinc, S.	2014	retrospective study	SSc	N	76	45	50.44 ± 13.21	46.52 ± 13.16	14/62	11/34	8
Wu, Y.	2016	retrospective study	SLE	N	116	136	27.81 ± 32.73	28.5 ± 12.75	19/97	25/111	8
Yolbas, S.	2016	retrospective study	BD	6.9 ± 5.9	53	55	37.4 ± 10.9	45.1 ± 13	20/33	11/44	8
			RA	8.2 ± 7.9	91	55	51.7 ± 14.5	45.1 ± 13	15/76	11/44	
			SLE	4.6 ± 5.2	51	55	33 ± 9.6	45.1 ± 13	4/47	11/44	
Zengin, O.	2016	retrospective study	RA	N	317	104	44.33 ± 10.41	43.16 ± 11.69	206/111	68/36	7

F: female; M: male; RA: rheumatoid arthritis; SLE: systemic lupus erythematosus; BD: Behçet’s Disease; AS: ankylosing spongdylitis; SD: standard deviation; NOS: Newcastle-Ottawa scale.

### Analysis

#### The relationship between NLR and ARDs

**Figure 2:**
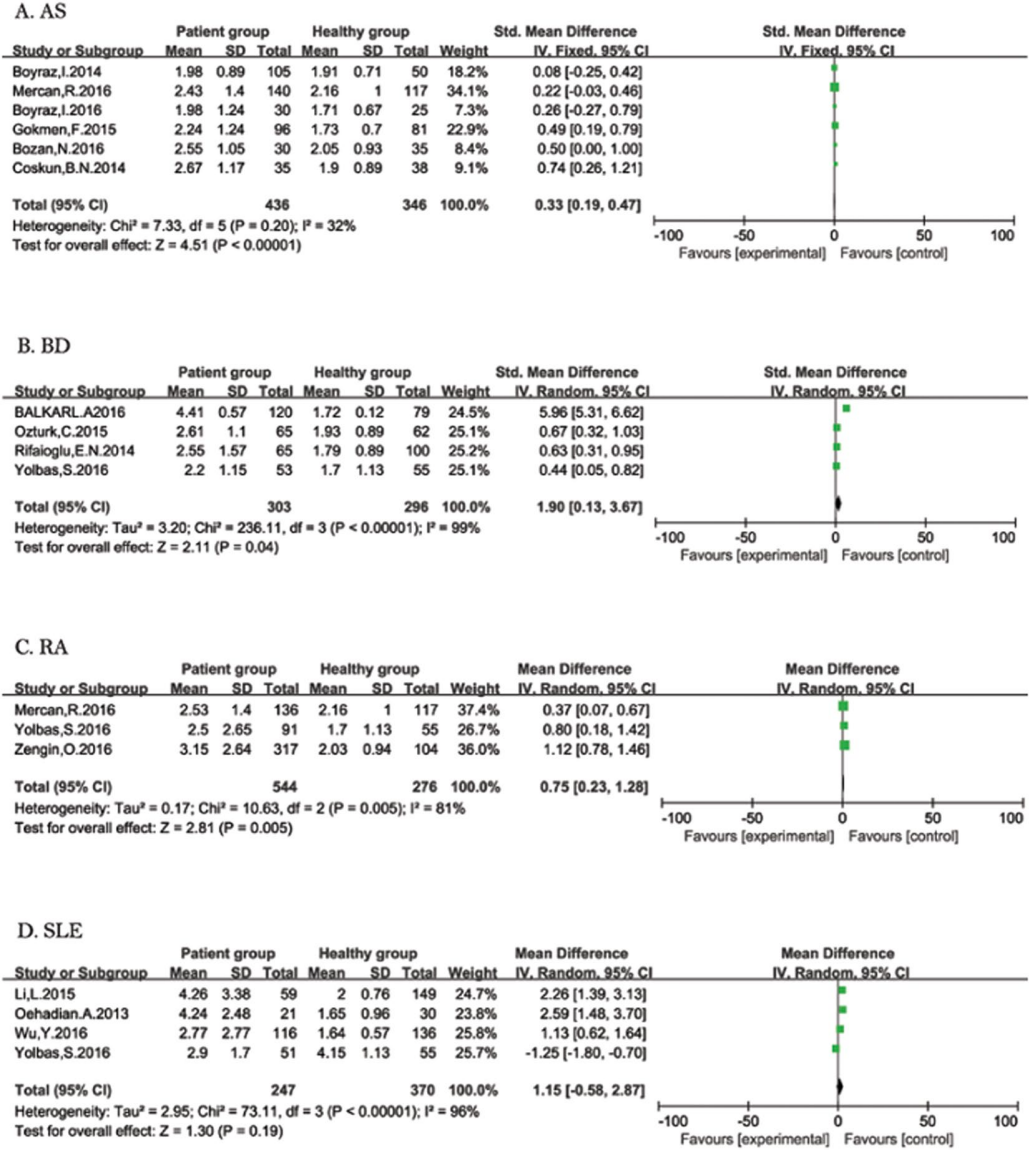
Forest plot of relationship between NLR and ARDs. AS (**A**), BD (**B**), RA (**C**), and SLE (**D**).

**AS:** Six studies were included in the forest plot (Fig. 2A). Fixed-effect meta-analysis was performed since significant heterogeneity was not present (I^2^ = 46%, *P* = 0.1), which showed that NLR was increased in patients with AS when compared with the healthy control group (SMD = 0.33; 95% CI: 0.19 to 0.47).

**BD:** Four studies were included in the forest plot (Fig. 2B). Random-effect meta-analysis was applied because of the significantly heterogeneity (I^2^ = 99%, *P* < 0.1). The result demonstrated that NLR was increased in patients with BD (SMD = 1.90; 95% CI: 0.13 to 3.67).

**RA:** Three studies showed the relationship between NLR and RA (Fig. 2C). The heterogeneity tests suggested significant heterogeneity (I^2^ = 81%, *P* < 0.1). Random-effect meta-analysis was applied since the heterogeneity. The result showed that NLR was higher in patients with RA compared to controls (SMD = 0.75; 95% CI: 0.23 to 1.28).

**SLE:** Four studies had data for SLE (Fig. 2D). Heterogeneity analysis showed I^2^ = 96%, and *P* < 0.1. Random-effect meta-analysis was applied since the heterogeneity. No association between NLR and SLE was observed (SMD = 1.15; 95% CI: −0.58 to 2.87).

#### The relationship between PLR and ARDs

**Figure 3:**
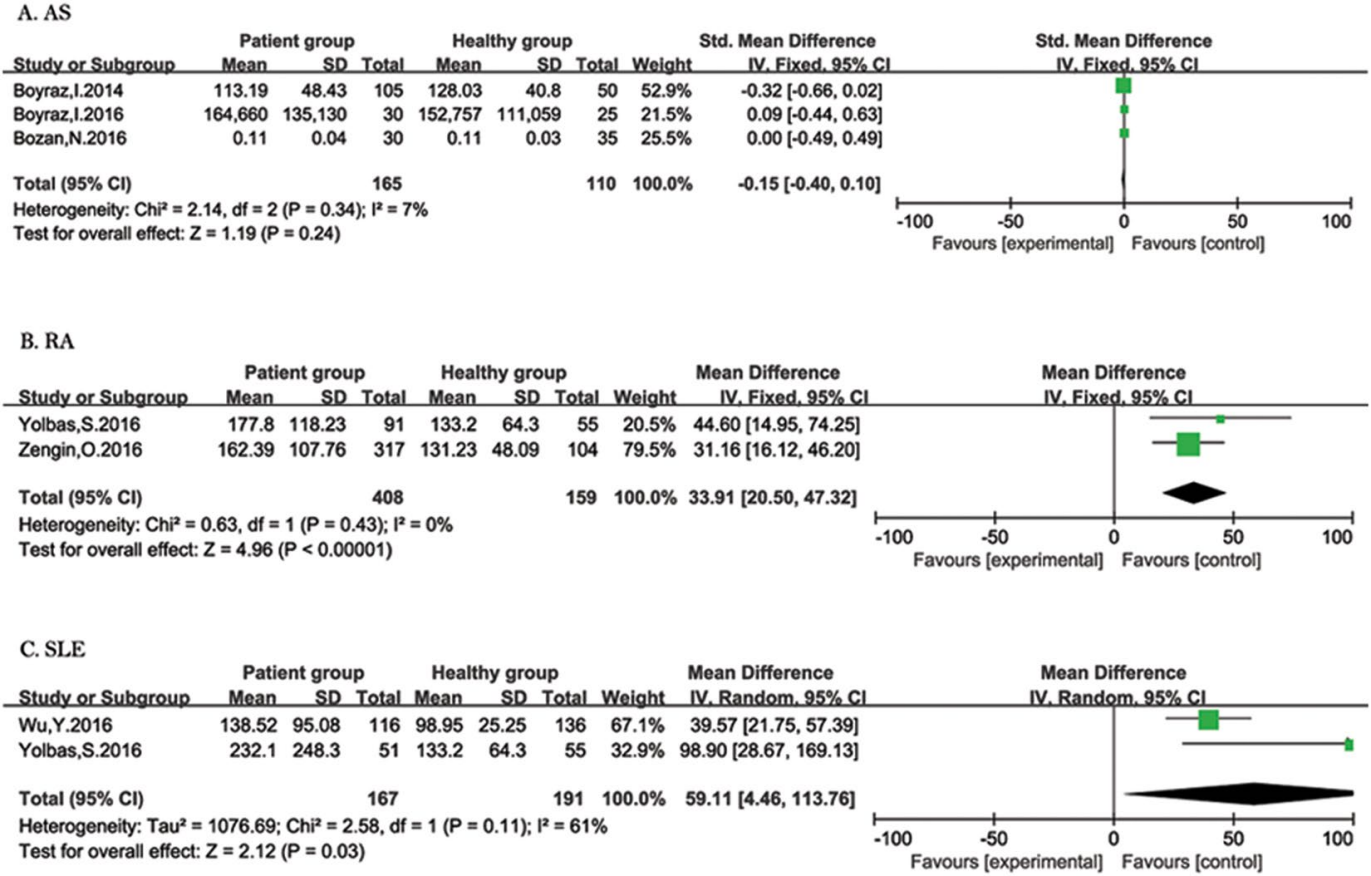
Forest plot of relationship between PLR and ARDs. AS (**A**), RA (**B**), and SLE (**C**).

**AS:** Three studies reported patients with AS (Fig. 3A). Heterogeneity analysis showed I^2^ = 7%, and *P* = 0.34 and fixed-effect meta-analysis was applied. No association between PLR and RA was observed (SMD = −0.15; 95% CI: −0.40 to 0.10).

**RA:** In terms of PLR and RA, the combined results from two studies showed that PLR was increased in patients with RA compared to healthy people (SMD = 33.91; 95% CI: 20.50 to 47.32) (Fig. 3B). The results of heterogeneity analysis were I^2^ = 0%, and *P* = 0.43 and fixed-effect meta-analysis was performed.

**SLE:** Twostudies reported patients with SLE (Fig. 3C). Heterogeneity analysis showed I^2^ = 61% and *P* = 0.11, which was severe; thus, random-effect meta-analysis was applied since the heterogeneity. There was an association increase in PLR in patients with SLE compared to healthy people (SMD = 59.11; 95% CI: 4.46 to 113.76).

#### The relationship between MPV and ARDs

**Figure 4:**
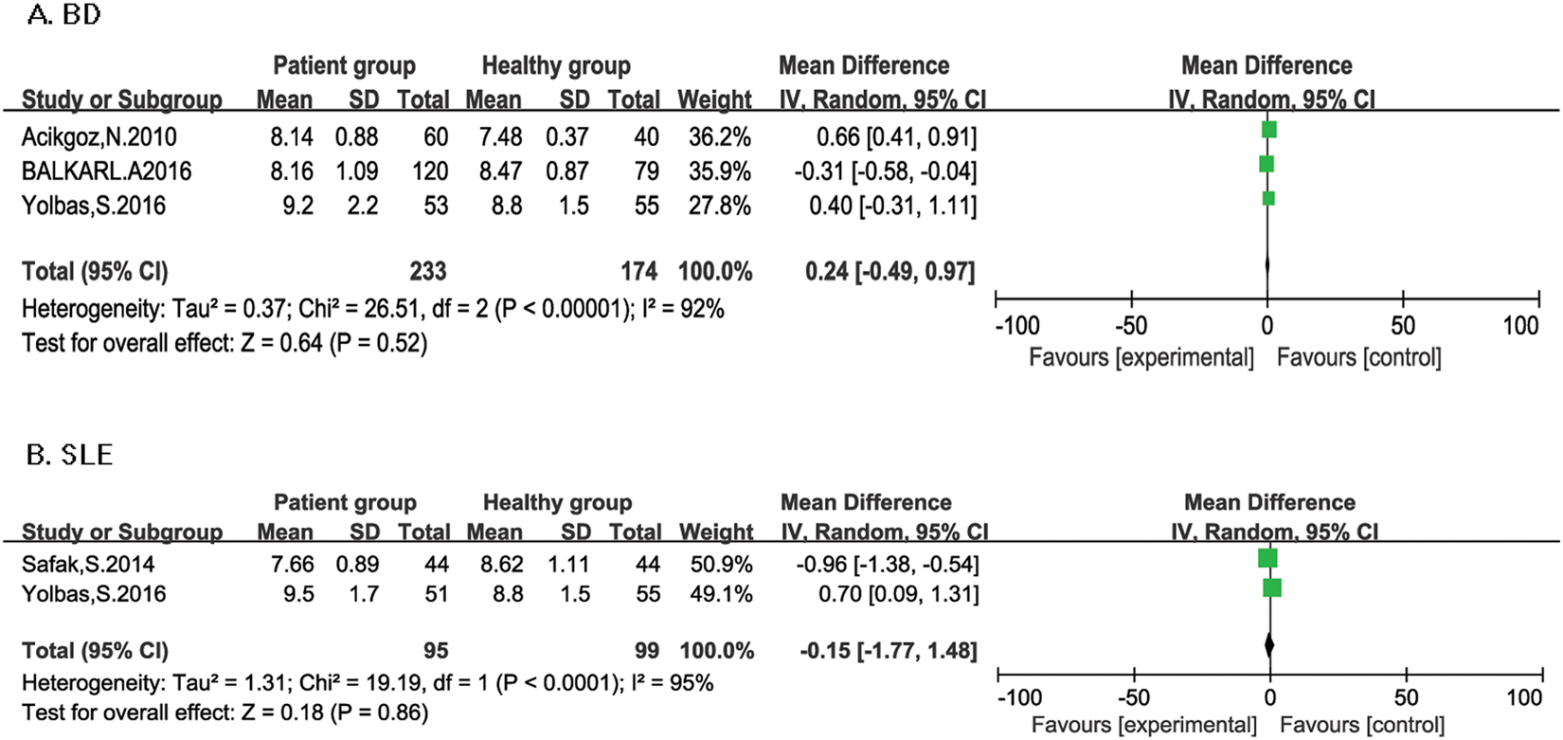
Forest plot of relationship between MPV and ARDs. BD (**A**), and SLE (**B**).

**BD:** Three studies reported patients with BD (Fig. 4A). Heterogeneity analysis showed I^2^ = 92%, and *P* < 0.1 and random-effect meta-analysis was applied. MPV was found to have no significant association with BD (SMD = 0.24; 95% CI: −0.49 to 0.97).

**SLE:** Two studies reported patients with SLE (Fig. 4B). Random-effect meta-analysis was performed since significant heterogeneity was found (I^2^ = 95%, *P* < 0.1). No association between PLR and RA was observed (SMD = −0.15; 95% CI: −1.77 to 1.48).

#### The relationship between RDW and ARDs

**Figure 5:**
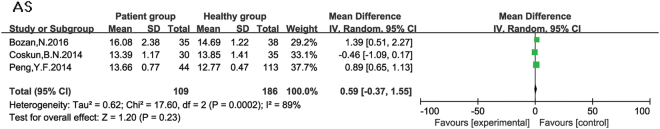
Forest plot of relationship between RDW and ARDs.

**AS:** ALL three studies reported RDW were about AS (Fig. 5). A relationship was not observed between RDW and AS (SMD = 0.59, 95% CI: −0.37, 1.55). Heterogeneity analysis showed I^2^ = 89%, and *P* < 0.1 and random-effect meta-analysis was applied.

### Sensitivity analysis and Publication bias

**Figure 6:**
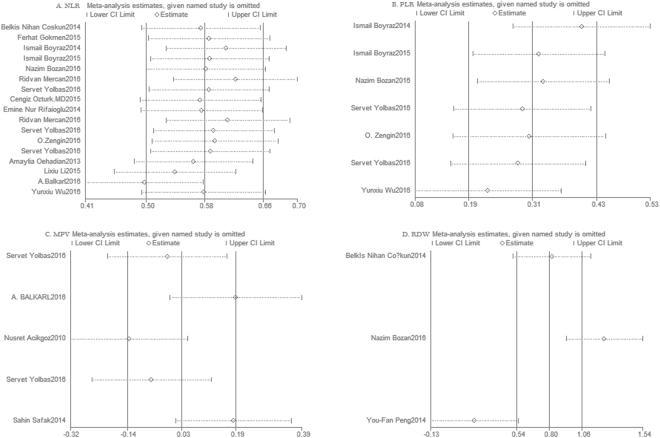
Sensitivity analysis of included studies. NLR and ARDs (**A**), PLR and ARDs (**B**), MPV and ARDs (**C**), and RDW and ARDs (**D**).

**Figure 7:**
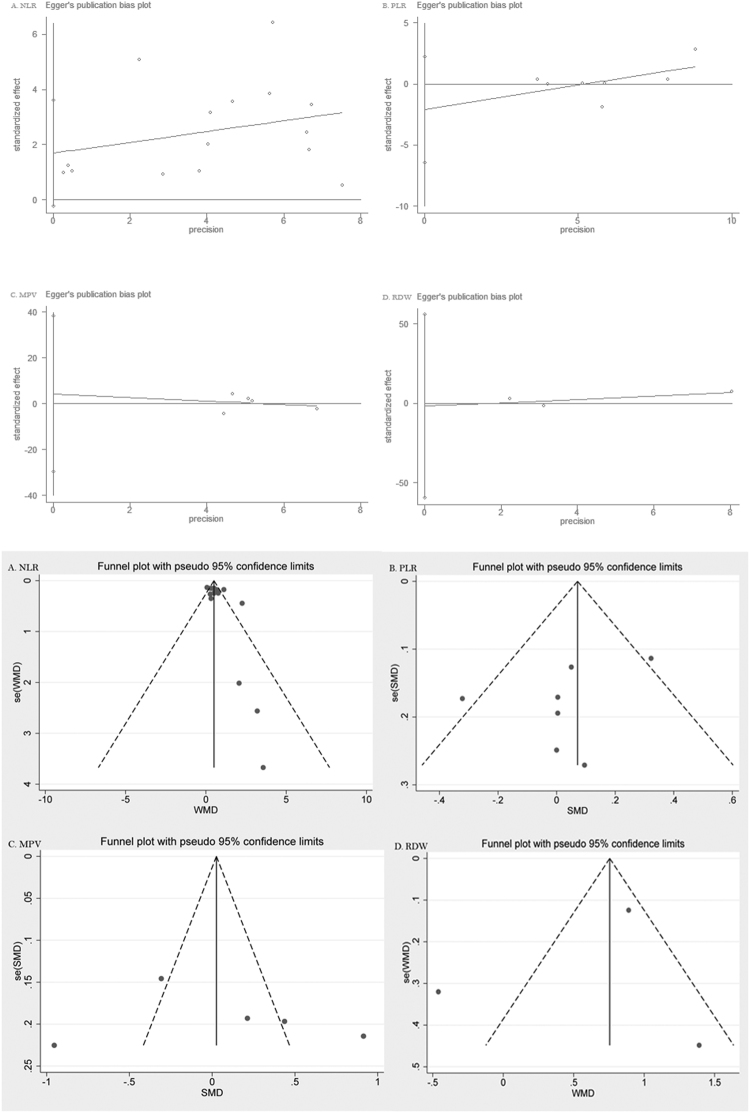
Funnel plot and Egger’s test evaluating the publication bias of studies. NLR and ARDs (**A**), PLR and ARDs (**B**), MPV and ARDs (**C**), and RDW and ARDs (**D**).

Sensitivity analysis was conducted by removal of one study every time from pooled analysis did not change significantly results. The results are shown in Fig. 6. Funnel plot and Egger’s test were performed to test publication bias. As shown in Fig. 7, the results indicated no evidence of publication bias.

## Discussion

As major inflammation markers, CRP, ESR, and white blood cell count (WBC) are commonly used for evaluating the activity of autoimmune rheumatic diseases. Recently, the hematological indices like NLR, PLR, MPV, and RDW have been reported as indicators of ADRs in some studies, since the inflammatory process in ARDs causes changes in the number, shapes, and sizes of peripheral blood cells. The hematological indices have been widely used as indicators for evaluation prognosis, and have been demonstrated that theyare associated with the severity of inflammation in diseases like inflammatory diseases, chronic renal failure, cardiovascular disease, diabetes mellitus, hypertension, and malignancies.

Neutrophils, lymphocytes and platelets have a significant place in the control of inflammation. As important regulators of innate and adaptive immune responses, neutrophils perform critical function and participate in the process of ARDs, including presenting antigen, regulating the activity of other cell types, and destroying tissue directly^23, 24^. Moreover, in the pathogenesis of inflammatory and immunity, cytokines, and some mediators such as tumor necrosis factor (TNF), interleukin-6 (IL-6), interleukin-7 (IL-7), interleukin-12 (IL-12) play prominent role. Circulating blood cell quantity and composition are involved in the production process of these cytokines and mediators. Lymphocytes are associated with ARDs since aberrant lymphocytes signaling leading to the pathogenesis autoimmunity, the activation of lymphocytes take response to chronic inflammation and damage on structure and function^25, 26^. By providing early signals to immune cells, active platelets are also source of inflammatory mediators and facilitate the secretion of inflammatory cytokines. Platelets play an active integral role in innate and adaptive immunity. In disease state, platelet microparticles releasing from thrombosis sites activate adaptive immune cells leading to antibody synthesis and alter lymphocyte activities, therefore, an immune response is simulated. The shape and size are changed when platelets are activated since larger platelets contain more enzymes and stronger ability of metabolism^21, 27^. NLR, PLR, MPV, and RDW were frequently used as inflammatory markers in some disease, while they were revealed just during the recent years for the ARDs.

To our knowledge, this is the first meta-analysis focusing on the relationship between hematological indices and ARDs. In our study, increasing NLR was found in patients with AS, BD, and RA. PLR was also found increased in patients with RA and SLE. We failed to demonstrate the relationship between MPV, RDW and ARDs. The results suggest that NLR and PLR might be used as new good biomarkers for RA and SLE.

NLR is an easily calculated, reproducible, cost-effective, and available marker of systemic inflammation. Neutrophil and lymphocyte count could be obtained from the complete blood count (CBC), which is a routine laboratory test in patients with autoimmune rheumatic disease for the monitoring the state and progress of disease. Moreover, NLR has been demonstrated as prognostic marker for cancers, such as urinary cancers^28^, lung cancer^29^, esophageal cancer^30^, and gynecologic cancers^31^. Similar to NLR, PLR is also an inflammatory index in some disease. Higher platelet and lower lymphocyte counts are associated with adverse clinic-pathologic features in some malignancies and chronic diseases.

However, several limitations in our meta-analysis should be acknowledged. Firstly, although we used random effect model, obvious heterogeneity between studies was observed, this may attributed to different characteristics of each studies (age, ethnicity, duration of disease, activity of disease, environmental factors), different quality of different research, different detecting instrument, and different experimental designs. The number of included studies limited the application of subgroup analysis and meta-regression analysis. Secondly, number of included studies was insufficient. Thirdly, due to the limited original data, the association between hematological indices and other rheumatic diseases such as dermatomyositis, psoriatic arthritis could not be explored. Fourthly, samples in the included studies were measured from peripheral blood, but not from the site of inflammation (such as synovial fluid for RA).

In conclusion, this meta-analysis indicated that NLR has a strong association with AS, BD, and RA. PLR was also related to RA and SLE. NLR and PLR could be recommended as inexpensive diagnostic biomarkers for rheumatic diseases. However, further large high quality investigations should be conducted to understand relationship between hematological indices and rheumatic diseases better.

## Methods

### Search strategy

This meta-analysis was conducted in accordance with Preferred Reporting Items for Systematic Review and Meta-analyses (PRISMA) guidelines (see Supplementary Table S1). A comprehensive literature search using the online databases of PubMed, Embase, and Web of Science was performed by two reviewers to identify relevant studies, the mean search terms included: “neutrophil*” “lymphocyte*”, “platelet–lymphocyte ratio”, “Red blood cell distribution width”, “mean platelet volume”, “hematological” and “rheumatic disease”, “rheumatoid arthritis”, “systemic lupus erythematosus”, “ankylosing spongdylitis”, “Sjögren’s syndrome”, “Behçet’s disease”, “systemic sclerosis”, the last search was updated to April30, 2017. References in the retrieved articles were also explored for potential relevant studies.

### Inclusion and exclusion criteria

Criteria for selecting the subjects were as follows: (1) Enrolled patients included were diagnosed with ARDs clearly; (2) observational studies; (3) subjects in the control group were healthy people; (4) original data were available; (5) English language full-text publication.

The exclusion criteria were as follows: (1) data were not available or abstract only; (2) animal studies, case reports, or letters to editors; (3) duplicated studies.

### Data extraction and Quality assessment

The following data were extracted after full-text articles reviewing by two reviewers: name of first author, title of article, publication year, total number of patients and controls, sex, mean age, disease studied, disease duration time, details of NLR, PLR, RDW, MPV. Two reviewers independently extracted data from articles, in cases of disagreement, consensus was reached by discussion. Included studies were evaluated using the Newcastle-Ottawa scale (NOS) which consists of eight items within three domains: selection, comparability, and exposure. Any discrepancy was discussed by the two authors.

### Statistical analysis

Standardized mean difference (SMD) was calculated with confidence interval (CI) of 95% for continuous variables. Statistical heterogeneity among studies was measured by Cochrane Q test (χ^2^) and I^2^ statistic: The Q test was used to test heterogeneity, and the I^2^ statistic was used to quantify the inconsistency: *P* < 0.1 for the Q test and I^2^ > 50% for the I^2^ statistic were appliedas significant heterogeneity of outcomes, in which case a random effect model was used to pool the data. Otherwise, the fixed effect model was adopted for analysis. When heterogeneity was present, possible sources were investigated via sensitivity analysis: each study dataset was dropped one at a time to detect the influence of each individual study. Funnel plot and Egger’s linear-regression test were performed to evaluatedpotential publication bias, those with P < 0.05 were considered to have publication bias. Statistical analyses were conducted using the STATA 12.0 software (Stata Corporation, College Station, TX, USA) and RevMan 5.3 (Cochrane Information Management Systems). A *P* < 0.05 was considered as statistically significant.

## Electronic supplementary material

Supplementary Information

Dataset 1
